# Initiating heavy-atom-based phasing by multi-dimensional molecular replacement

**DOI:** 10.1107/S2059798315022482

**Published:** 2016-03-01

**Authors:** Bjørn Panyella Pedersen, Pontus Gourdon, Xiangyu Liu, Jesper Lykkegaard Karlsen, Poul Nissen

**Affiliations:** a Centre for Membrane Pumps in Cells and Disease, Danish National Research Foundation, Department of Molecular Biology and Genetics, Aarhus University, Gustav Wieds Vej 10C, DK-8000 Aarhus, Denmark

**Keywords:** experimental phasing, molecular replacement, heavy-atom substructure

## Abstract

To obtain an electron-density map from a macromolecular crystal the phase problem needs to be solved, which often involves the use of heavy-atom derivative crystals and concomitant heavy-atom substructure determination. This is typically performed by dual-space methods, direct methods or Patterson-based approaches, which however may fail when only poorly diffracting derivative crystals are available. This is often the case for, for example, membrane proteins. Here, an approach for heavy-atom site identification based on a molecular-replacement parameter matrix (MRPM) is presented. It involves an *n*-dimensional search to test a wide spectrum of molecular-replacement parameters, such as different data sets and search models with different conformations. Results are scored by the ability to identify heavy-atom positions from anomalous difference Fourier maps. The strategy was successfully applied in the determination of a membrane-protein structure, the copper-transporting P-type ATPase CopA, when other methods had failed to determine the heavy-atom substructure. MRPM is well suited to proteins undergoing large conformational changes where multiple search models should be considered, and it enables the identification of weak but correct molecular-replacement solutions with maximum contrast to prime experimental phasing efforts.

## Abstract 2

A strategy is presented to set up an *n*-dimensional molecular-replacement parameter matrix (MRPM) search using anomalous difference Fourier maps from related data sets to uncover weak, but correct, molecular-replacement solutions for heavy-atom substructure determination and subsequent experimental phasing.

## 1. Introduction   

To determine the structure of a macromolecular crystal, the phase problem must be solved. For isomorphous replacement and anomalous scattering methods (referred to as experimental phasing in this paper), phasing can be considered a two-step procedure in which the heavy-atom (HA) substructure is initially derived, after which the substructure is used to calculate phases for the entire macromolecular structure (Hendrickson, 1991 ▸; Dauter *et al.*, 2002 ▸). Knowing the substructure, reasonable experimental maps can often be generated from surprisingly weak data thanks to improvements in statistical phase probability calculations and density-modification procedures (Terwilliger, 2000 ▸, 2001 ▸; McCoy, 2002 ▸; Jenni *et al.*, 2006 ▸; Keller *et al.*, 2006 ▸; Maier *et al.*, 2006 ▸; McCoy & Read, 2010 ▸; Abrescia *et al.*, 2011 ▸; Li & Li, 2011 ▸; Liu *et al.*, 2011 ▸)

Typically, the heavy-atom substructure is found using Patterson-based methods, direct methods or frequently dual-space methods, which can combine Patterson-based seeding with direct methods and real-space steps (Hendrickson & Ogata, 1997 ▸; Weeks & Miller, 1999 ▸; Burla *et al.*, 2003 ▸; Grosse-Kunstleve & Adams, 2003 ▸; Sheldrick, 2008 ▸). Such heavy-atom site identification is nontrivial when only weak diffraction data of poor quality are available and is often complicated by crystal and data pathologies such as radiation damage and severe anisotropy (Skubák & Pannu, 2013 ▸; Bunkóczi *et al.*, 2015 ▸).

Molecular replacement (MR) is an alternative method for obtaining phase estimates. However, if the experimental data are of low resolution and low quality, the end result will be highly biased by the model (Read, 1986 ▸; DeLaBarre & Brunger, 2006 ▸), obscuring rebuilding and refinement of the proper target structure.

Nonetheless, MR is still useful in such difficult cases. By using molecular replacement at low resolution, an initial starting model, despite very low sequence identity, can generate phases which allow the identification of HA peaks through anomalous difference Fourier maps (de La Fortelle & Bricogne, 1997 ▸; McCoy & Read, 2010 ▸). After positioning of the heavy atom(s), the model-biased MR phases can in principle be discarded and phase calculation and improvement can be conducted using traditional methods. For examples, see Pedersen *et al.* (2007 ▸) and Mourão *et al.* (2014 ▸).

Here, we present a systematic expansion of this approach that we developed for the structure determination of the copper-transporting P-type ATPase CopA (Gourdon, Liu *et al.*, 2011 ▸). The identification of heavy-atom sites in CopA HA-derivative data turned out to be highly challenging. While an extensive effort was put into the generation of improved derivative and native crystals, a strategy to systematically screen MR parameters was developed that we have dubbed molecular-replacement parameter matrix (MRPM) search, since traditional approaches failed to facilitate structure determination and refinement.

## 2. Materials and methods   

### 2.1. Sample description   

CopA is a copper-exporting membrane protein that belongs to the well studied family of primary transporters known as P-type ATPases (Møller *et al.*, 1996 ▸; Axelsen & Palmgren, 1998 ▸; Palmgren & Nissen, 2011 ▸). This family has a transmembrane (M) domain with a common core of six transmembrane (TM) helices, and three soluble domains, known as the A, N and P domains (Morth *et al.*, 2011 ▸). Crystallization of a CopA family member from *Legionella pneumophila* (LpCopA) resulted in crystals that diffracted to about 3 Å resolution in the best case but suffered from severe non-isomorphism between most data sets (Supplementary Table S1; Gourdon, Andersen *et al.*, 2011 ▸; Gourdon, Liu *et al.*, 2011 ▸).

### 2.2. Method description   

The identification of a correct MR solution is not trivial when the search model and/or experimental data are of poor quality. The use of various high-resolution data cutoffs and estimated root-mean-square coordinate errors (r.m.s.) of the search model should be explored, and search-model completeness *versus* correctness should be ensured (Pedersen *et al.*, 2010 ▸; Oeffner *et al.*, 2013 ▸). If conformational flexibility of the target is possible, different conformational states should be tested as well.

Here, we include a number of model conformations and search parameters in a systematic expansion to explore a large MR parameter space. Since the end goal is to identify consistent HA peaks in a substructure determination, the numerous MR solutions are scored using this criterion and simultaneously the corresponding *Z*-score to help to distinguish correct solutions from noise.

### 2.3. Hardware and software   

The computer used was a regular Linux desktop computer [4× Intel Xeon CPU W3540 (2.93 GHz), 24 GB RAM]. A total of 397 CPU hours were used for this analysis. In real time, the calculations took 4 d 3 h 20 min.

All scripts were made using the Bourne shell (sh). Example scripts sufficient to perform a similar analysis are provided as Supporting Information. The programs used were *Phaser* (McCoy *et al.*, 2007 ▸), *PEAKMAX* (Winn *et al.*, 2011 ▸), *SCALEIT* (Howell & Smith, 1992 ▸), *FFT* (Ten Eyck, 1973 ▸), *SUPERPOSE* (Krissinel & Henrick, 2004 ▸), *PyMOL* (http://www.pymol.org) and *gnuplot* (http://www.gnuplot.info).

## 3. Results and discussion   

**Figure 1:**
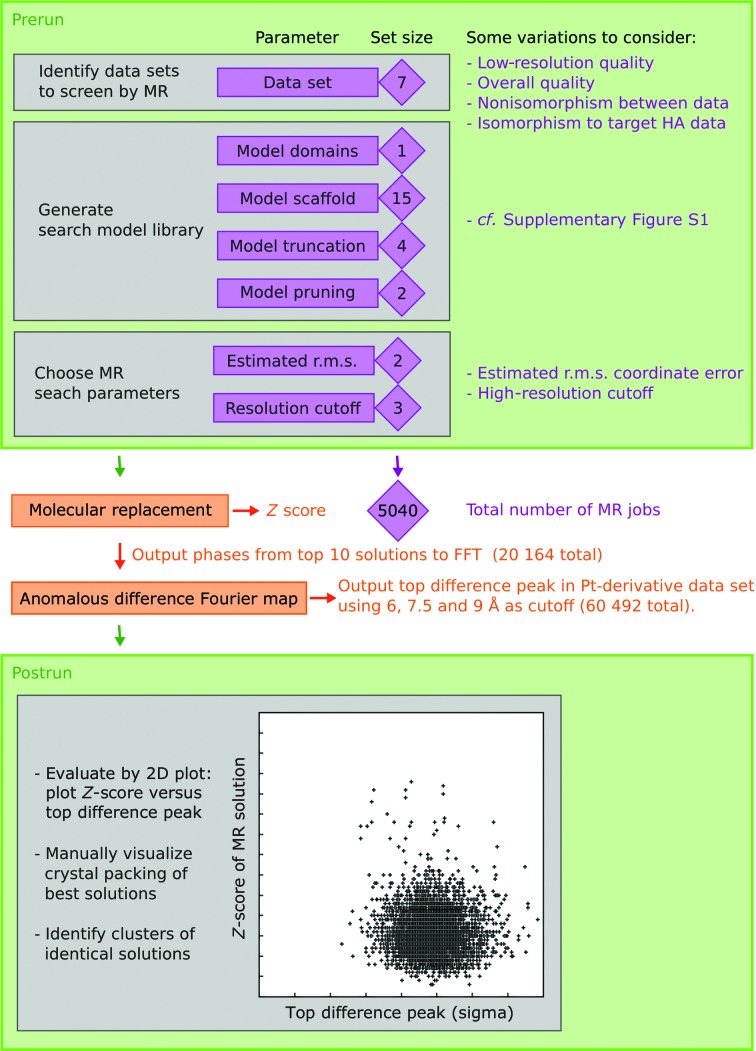
Overview of the MRPM search strategy. Prerun considerations (top green box) have to be made to identify parameters (dimensions) and sets of values to test for each parameter. The parameters and set size for each parameter shown here are specific for the CopA case. After each MR and FFT calculation, the result is plotted on a two-dimensional plot to identify clusters of MR solutions that both have a high *Z*-score and generate large difference peaks in the Pt-derivative data set.

A schematic representation of the MRPM strategy is shown in Fig. 1 ▸. Manually analyzing the heavy-atom derivative data sets collected, a K_2_PtCl_6_-derivative data set was identified to be our superior HA data set, *i.e.* that with the most significant anomalous difference signal, in this case extending to 5.5 Å resolution (Supplementary Table S2). A strategy was therefore designed to evaluate whether MR phases could identify significant anomalous difference peaks in this Pt-derivative data set.

### 3.1. Generation of the search-model library   

Several full-length P-type ATPase structures (predominantly of the rabbit sarcoplasmic reticulum Ca^2+^-ATPase 1a) are available in the Protein Data Bank (PDB), representing a library of conformational states that are characteristic of this protein family. We regard a search model as composed of a number of domains placed according to different scaffold structures representing conformational states. To further increase sampling, the domains are subjected to different truncations of loop regions or whole domains and pruning of the side-chain atoms, leading to sublibraries of related search models.

For scaffolds, 33 P-type ATPase structures were downloaded and an r.m.s. deviation matrix of the C^α^ atoms was calculated (Supplementary Table S3). Redundant scaffold structures were identified, resulting in 15 unique scaffolds with greater than 1 Å r.m.s. deviation from each other (Supplementary Table S4).

Structures of isolated A, N and P domains with high sequence identity to our LpCopA target were identified by *BLAST*. For the M domain, the six core TM helices of each of the 15 scaffolds were used. These four domains together cover ∼71% of the CopA sequence (Supplementary Table S5). Missing parts of CopA included the heavy-metal binding domain and the two N-terminal TM helices; both are specific features of heavy-metal pumps and had unknown positions relative to the scaffolds.

The four domains were placed by superposition into the 15 scaffolds, resulting in 15 starting models representing the conformational variability observed in the database of P-type ATPase structures (Supplementary Fig. S1, steps 1 and 2; Supplementary Fig. S2).

To compensate for potential domain flexibility or domain-structure errors, we included three truncated versions for each starting model (A, N and M domain removed, respectively; Supplementary Fig. S1, step 3), and these four versions of each starting model were generated in two forms: either with all atoms included or pruned to polyalanines only (Supplementary Fig. S1, step 4). In total, the final library contained 120 different search models (Supplementary Tables S6–S9).

### 3.2. Setting up the MR parameter-matrix search   

Six native data sets were selected, based on criteria such as good quality of the low-resolution data, highest obtained resolution and best scaling overall to the Pt-derivative data set (Supplementary Table S2). Assuming one monomer per asymmetric unit, the solvent content was estimated to be about 62%, which is typical of membrane-protein crystals.

Based on previous experience with MR and low-quality data (Pedersen *et al.*, 2010 ▸), we tested different values for the expected r.m.s. coordinate error (2 or 3 Å) and high-resolution limits of the data (4, 6 and 8 Å), while leaving other parameters constant.

The final parameter matrix systematically combined these six search-parameter setups with seven data sets and 120 search models, parsing a total of 5040 MR searches for analysis (Fig. 1 ▸). As the correct solution was expected to be weak, the ten best final solutions from each run were saved and evaluated. Postrun analysis shows that a total of 20 164 suggested MR solutions were output from the 5040 MR searches.

### 3.3. Evaluation   

An anomalous difference Fourier map of the Pt-derivative data set was calculated for each of the 20 164 MR solutions. Peaks are expected to be weak in such maps and very sensitive to the resolution cutoff. To adress this, three cutoff values (6, 7.5 and 9 Å) were used. The highest anomalous difference peak for each of the 60 492 maps was identified and plotted (peak height in σ units) as a function of the *Z*-score of the input MR solution.

**Figure 2:**
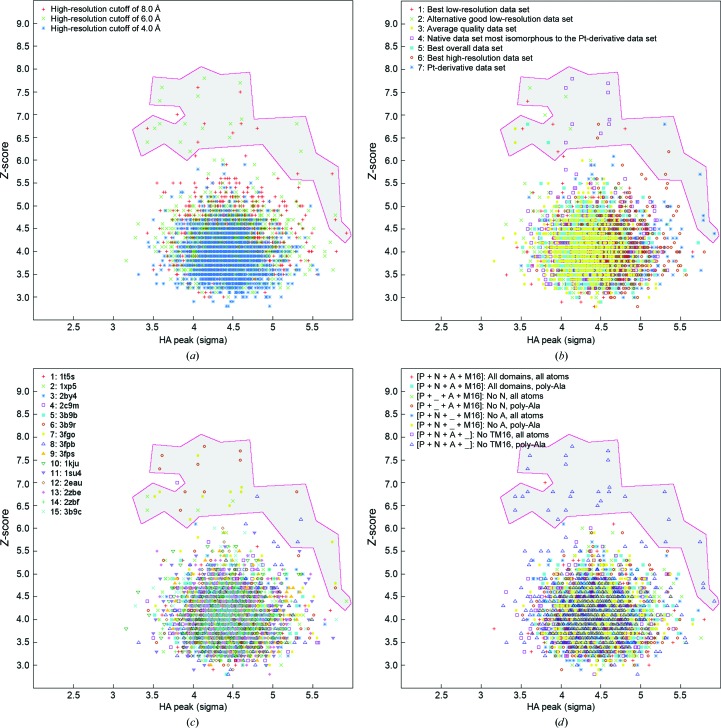
Two-dimensional plot of the result of the MR parameter search. All solutions are plotted as a function of *Z*-score and corresponding highest difference peak in the Pt-derivative data set. The grey area highlights the MR solutions that turned out to be identical and correct. (*a*) High-resolution cutoff. (*b*) Data set used. (*c*) Scaffold used. The PDB code is noted. (*d*) Truncation and pruning used.

The majority of MR solutions had low *Z*-scores (<5.5) and did not give rise to significant difference peaks (<5σ), indicating failed MR searches. However, a number of favourably scored MR solutions were apparent and through evaluation according to the various screened parameters a tantalizing pattern emerged (Fig. 2 ▸).

A broad selection of top-scoring solutions were manually analyzed and we found that 30 of these were virtually identical and all identified the same difference peak (highlighted in Fig. 2 ▸). All of these required the exclusion of high-resolution data, a scaffold with an ‘outward-facing occluded’ conformation and a polyalanine model excluding the M domain. Depending on the MR data set used, the parameters would either give a notable *Z*-score or a notable difference peak.

The phases from MR using these parameters allowed the determination of two initial positions of Pt atoms leading to experimental phases and allowing structure determination to proceed (Gourdon, Liu *et al.*, 2011 ▸).

The best MR solution as evaluated by *Z*-score alone (*Z*-score 7.8) was a correct solution, but the Pt peak calculated using the phases from this particular solution was insignificant (4.14σ), likely owing to non-isomorphism to the Pt-derivative data set. We must emphasize that even if by serendipity the best possible selection of parameters tested here had been used in a single MR run, the result would still not be sufficiently clear in its own right to indicate a correct solution. Only by comparing a large number of solutions did a consistent picture emerge, which lent confidence to the subsequent analysis. One solution, for example, had an MR *Z*-score of 7.0, and another produced an anomalous difference peak at 5.79σ, which both appeared to be promising indications of a successful solution but which both also turned out to be wrong (Fig. 2 ▸).

## 4. Concluding remarks   

For CopA, the molecular-replacement parameter matrix search presented here was our workaround to initiate phasing for a structure determination that was plagued by weak diffraction properties and poor crystal-to-crystal isomorphism. We believe that the MRPM search strategy is of general interest for numerous projects with analogous challenges as well as in more standard applications. It can easily be extended to use more or different dimensions than those presented here. Employing an array of different domains (for example, domains solved from different organisms) is one example. Testing more data sets and using alternative methods of search-model pruning, as well as full mutagenesis to the target sequence or the creation of mixed models, are other obvious possibilities [using, for example, *CHAINSAW* (Stein, 2008 ▸) and *Sculptor* (Bunkóczi & Read, 2011 ▸)]. Furthermore, multiple derivative data sets could easily be employed to identify consistent sets of different HA peaks.

An ever-increasing number of programs target the phase problem in different ways, and our choice of programs is not necessarily the best one for any given case. Instead, we wish to emphasize the general value of systematic sampling for difficult cases, and this may also include different programs or approaches. For instance, one could try using log-likelihood-gradient completion in *Phaser* to find the heavy-atom sites (Read & McCoy, 2011 ▸) instead of calculating anomalous difference maps, or for relatively good-resolution data use *SHELXE* to reduce model bias and obtain an indication of whether MR solutions are correct without using any derivative data and experimental phasing (Thorn & Sheldrick, 2013 ▸). Keeping in mind the advent of improved protein-folding algorithms (Qian *et al.*, 2007 ▸; Rigden *et al.*, 2008 ▸; DiMaio *et al.*, 2011 ▸), generic search models (Strop *et al.*, 2007 ▸) and automated procedures (Keegan & Winn, 2007 ▸; Stokes-Rees & Sliz, 2010 ▸), as well as pipelines using large numbers of input search models (Bibby *et al.*, 2012 ▸; Sammito *et al.*, 2013 ▸), the importance of testing different conformational states is accentuated by the work presented here, and it emphasizes an aspect of modelling that is not currently addressed by *in silico* modelling.

Traditionally, crystallographic structure determination has proceeded through either experimental phasing or molecular replacement. MRPM is a hybrid approach in which heavy-atom derivative-based scoring is used to distinguish proper MR solutions that conversely determine the heavy-atom substructure to initiate experimental phasing. As another example of this, *phenix.mr_rosetta* takes a set of potential MR solutions and rebuilds each of these using *Rosetta* force fields to obtain the correct solution (Terwilliger *et al.*, 2012 ▸).

In general, systematic MR searches should be strongly preferred over single MR runs, using for instance an MRPM strategy as described here in conjunction with powerful combinatory approaches such as *MrBUMP* and *Wide Search Molecular Replacement* (Keegan & Winn, 2007 ▸; Stokes-Rees & Sliz, 2010 ▸). Even if derivative data sets are not available, a systematic search is more likely to help to identify a correct solution and distinguish it from false positives when only data of limited quality are available.

## Supplementary Material

Supporting Information.. DOI: 10.1107/S2059798315022482/ba5231sup1.pdf
