# Supplementary material for: Initiating heavy-atom-based phasing by multi-dimensional molecular replacement
Source: Acta Crystallogr D Struct Biol. 2016 Mar 1;72(Pt 3):440–5. doi: 10.1107/S2059798315022482 (PMC4784675; doi:10.1107/S2059798315022482)
Supplement: Supplementary file 1 [file d-72-00440-sup1.pdf]

STRUCTURAL BIOLOGY

Volume 72 (2016)

Supporting information for article:

Initiating heavy-atom-based phasing by multi-dimensional molecular replacement

Bjørn Panyella Pedersen, Pontus Gourdon, Xiangyu Liu, Jesper Lykkegaard Karlsen and Poul Nissen

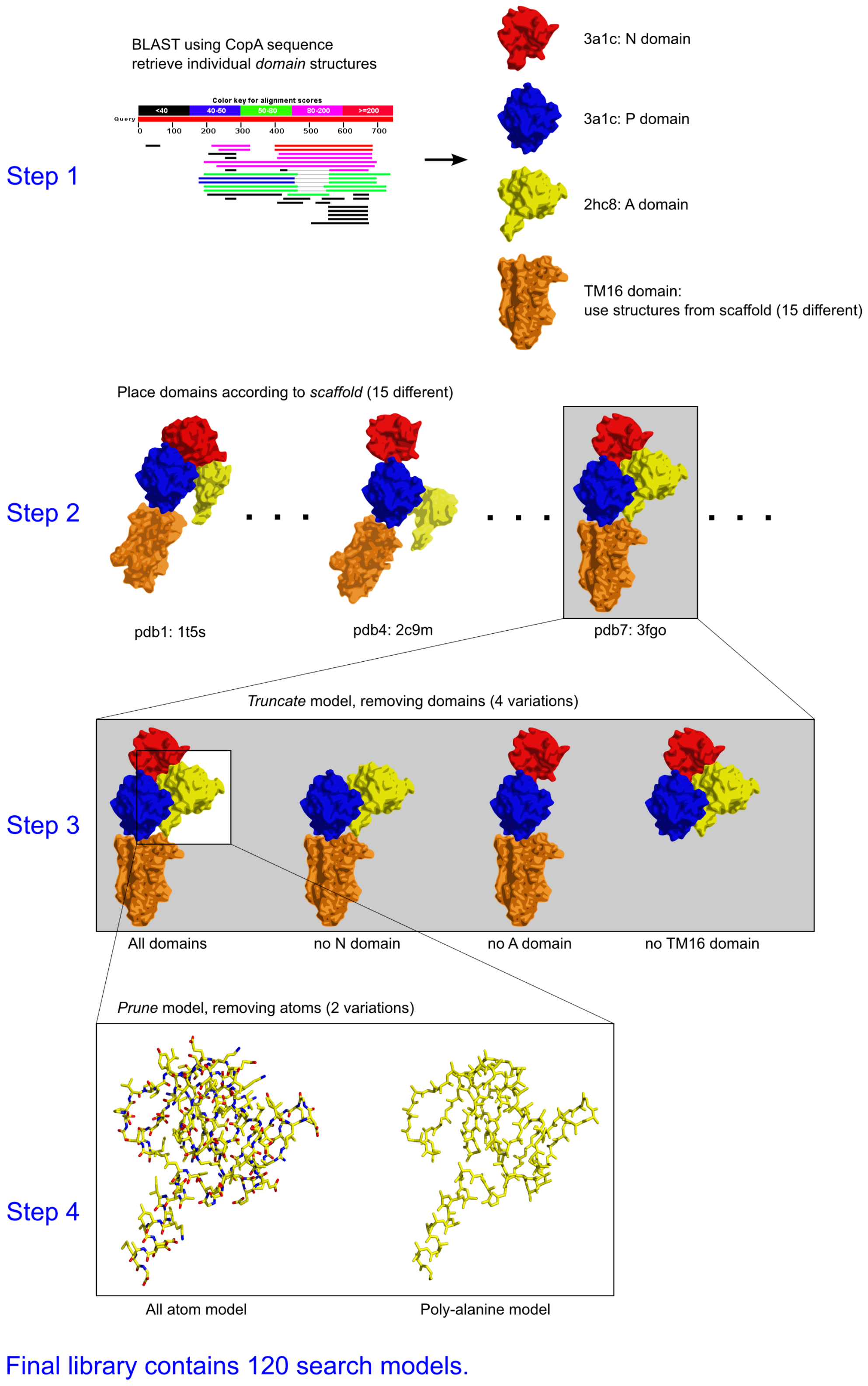

Supplementary Figure S1. Generation of the search model library. Generation of the library is divided into 4 steps. *Step 1:* Identify the domains to use. *Step 2:* Identify the scaffolds used to place the domains into representing different possible conformations and place the domains into these scaffolds. *Step 3:* Identify and generate a number of truncations removing different domains, since one incorrectly placed domain can make the difference between success and failure. *Step 4:* Prune the atoms of the models to generate variations. In this particular case only two different pruning schemes were used: all atom or reduction to poly-alanine.

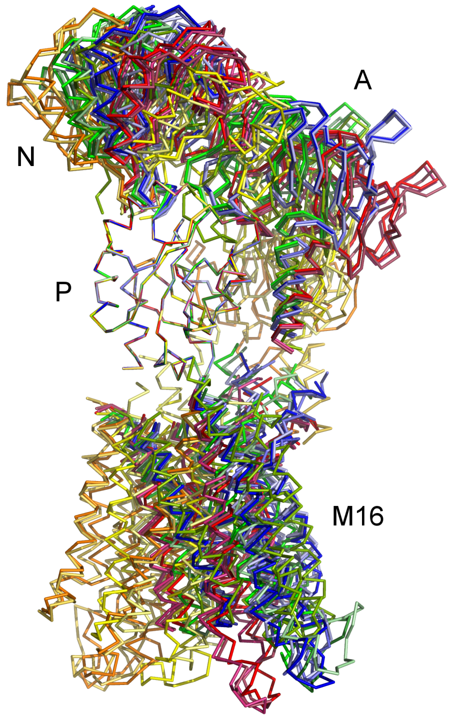

Supplementary Figure S2. Superposition of the 15 starting models after step 2 in Supp. Figure 1. All models are superposed on the P domain and each model has a distinct color. The conformational variation obtained by using different scaffolds is evident. The functional cycles of P-type ATPases is characterized by four principal conformations (Møller et al., 2010). The colors are chosen to emphasize that the models fall into these classes: Red shades are the Occluded outwards facing forms. Blue shades are the occluded transition state forms. Green shades are the open outward facing forms. Yellow shades are the inward facing forms.

Supplementary Table S1. Non-isomorphism between datasets. Total R-factor on F by *SCALEIT* from 50-6 Å. Dataset 7 is the Pt-derivative dataset (cf. Supplementary Table S2).

**R(cross)**	**Dataset 1**	**Dataset 2**	**Dataset 3**	**Dataset 4**	**Dataset 5**	**Dataset 6**	**Dataset 7**
**Dataset 1**	-	0.078	0.342	0.192	0.094	0.213	0.355
**Dataset 2**	0.078	-	0.367	0.152	0.138	0.259	0.335
**Dataset 3**	0.342	0.367	-	0.407	0.329	0.300	0.499
**Dataset 4**	0.192	0.152	0.407	-	0.243	0.338	0.297
**Dataset 5**	0.094	0.138	0.329	0.243	-	0.171	0.436
**Dataset 6**	0.213	0.259	0.300	0.338	0.171	-	0.439
**Dataset 7**	0.355	0.335	0.499	0.297	0.436	0.439	-

Supplementary Table S2. Dataset statistics.

	Dataset 1	Dataset 2	Dataset 3	Dataset 4	Dataset 5	Dataset 6	Dataset 7
Type	native	native	native	native	native	native	K2PtCl-derivative
Reason for inclusion in this analysis.	High quality at low resolution.	Alternative high quality at low resolution.	Average quality.	Native most isomorph to the Pt-dataset.	High quality overall.	Best high resolution.	Best heavy atom derivative.
**Overall statistics**							
Space group	$P2_12_12_1$	$P2_12_12_1$	$P2_12_12_1$	$P2_12_12_1$	$P2_12_12_1$	$P2_12_12_1$	$P2_12_12_1$
Cell dimensions							
a, b, c (Å)	44.21, 72.51, 328.92	44.12, 72.59, 330.18	43.77, 71.76, 324.05	44.72, 72.61, 329.53	44.53, 72.76, 329.65	43.87, 72.14, 327.28	45.03, 72.53, 329.25
α, β, γ (°)	90, 90, 90	90, 90, 90	90, 90, 90	90, 90, 90	90, 90, 90	90, 90, 90	90, 90, 90
Resolution	50-3.7 (3.8-3.7)[a]	50-4.0 (4.1-4.0)	50-3.7 (3.8-3.7)	50-4.1 (4.2-4.1)	50-3.55 (3.6-3.55)	50-3.4 (3.5-3.4)	50-5.5 (6.0-5.5)
Rsym (%)	15.4 (92.8)	13.7 (68.4)	32.2 (154.2)	12.1 (36.9)	14.4 (161.7)	10.7 (117.3)	10.9 (54.9)
Rmeas (%)[b]	16.6 (100.0)	15.1 (75.1)	34.8 (169.2)	15.0 (46.1)	15.4 (171.5)	11.9 (132.7)	12.5 (62.7)
Rmrgd-F (%)[c]	18.3 (74.5)	19.4 (72.3)	37.9 (156.6)	25.9 (69.6)	17.0 (109.9)	16.7 (116.3)	18.4 (63.3)
I/sig(I)	12.65 (2.05)	11.57 (2.41)	6.2 (1.00)	6.63 (2.52)	11.77 (1.46)	12.71 (1.55)	9.28 (2.65)
Completeness (%)	99.7 (99.9)	99.4 (99.5)	99.2 (97.2)	99.1 (99.8)	99.8 (99.3)	98.7 (94.9)	99.0 (98.4)
Redundancy	7.0 (7.2)	5.6 (5.7)	6.8 (5.1)	2.8 (2.7)	8.6 (8.8)	5.4 (4.3)	4.3 (4.2)
SigAno[d]	-	-	-	-	-	-	1.181 (0.805)
**High resolution quality**							
Rmrgd-F (4.1-4.2 Å) (%)	30.7	48.4	74.4	69.6	23.4	17.7	-
I/sig(I) (4.1-4.2 Å)	5.28	3.33	2.13	2.52	6.66	8.94	-
**Low resolution quality**							
Rmrgd-F (20-30 Å) (%)	1.2	1.1	2.1	3.2	2.0	2.0	3.8
I/sig(I) (20-30 Å)	66.69	56.45	33.93	20.33	38.80	48.02	20.68
**Pt-derivate signal**							
SigAno (15-50 Å)	-	-	-	-	-	-	2.980
SigAno (7-8 Å)	-	-	-	-	-	-	1.158

a: Values in parentheses are from the highest resolution shell.
b: R-meas = redundancy independent R-factor (intensities). (Diederichs & Karplus (1997), Nature Struct. Biol. 4, 269-275.)
c: Rmrgd-F = quality of amplitudes (F) in the scaled data set. (Diederichs & Karplus (1997), Nature Struct. Biol. 4, 269-275.)
d: SigAno = mean anomalous difference in units of its estimated standard deviation (|F(+)-F(-)|/Sigma). F(+), F(-) are structure factor estimates obtained from the merged intensity observations in each parity class (as calculated in XSCALE).

Supplementary Table S3. C-alpha r.m.s. deviation between the 32 initially identified Ca^{2+}-ATPase scaffolds. The code refers to the scaffolds pdb-id. Red notes r.m.s.d. below 1 Å.

	1T5S	1T5T	1XP5	2BY4	2C88	2C8K	2C8L	2C9M	3B9B	3B9R	3BA6	3FGO	3FPB	3FPS	2O9J	2OA0	1IWO	1KJU	1SU4	1VFP	1WPG	2AGV	2DQS	2EAR	2EAS	2EAT	2EAU	2ZBD	2ZBE	2ZBF	2ZBG
**1T5S**	0.00	0.18	4.56	3.63	3.58	3.64	3.70	2.14	3.90	4.74	0.46	4.53	4.88	3.85	4.89	3.81	3.69	4.25	2.29	0.83	4.58	3.76	3.76	3.53	3.38	3.43	3.84	0.69	4.27	4.31	4.88
**1T5T**	0.18	0.00	4.51	3.67	3.60	3.54	3.65	2.13	3.82	4.62	0.43	4.37	4.77	3.62	4.94	3.52	3.81	4.39	2.28	0.86	4.69	3.67	3.62	3.57	3.39	3.45	3.86	0.71	4.15	4.28	4.82
**1XP5**	4.55	4.57	0.00	2.65	2.79	2.74	2.75	3.44	3.48	1.54	4.50	1.50	1.34	2.84	1.33	2.84	2.72	2.79	2.39	4.45	1.10	2.74	2.71	2.75	2.78	2.63	2.83	4.60	2.83	2.64	0.97
**2BY4**	3.63	3.65	2.67	0.00	0.51	0.57	0.58	4.36	3.83	2.83	3.62	2.92	3.00	1.03	2.97	0.97	0.81	3.56	4.51	3.66	2.71	0.83	0.78	1.02	1.18	1.04	1.38	3.76	3.48	2.85	2.62
**2C88**	3.58	3.65	2.79	0.51	0.00	0.30	0.34	4.40	3.77	2.84	3.56	2.94	2.90	0.99	2.85	0.96	0.68	3.52	4.83	3.58	2.73	0.72	0.67	0.99	1.11	0.95	1.33	3.80	3.44	2.72	2.70
**2C8K**	3.70	3.54	2.72	0.57	0.30	0.00	0.32	4.29	3.66	2.83	3.56	2.98	2.89	1.00	2.86	0.96	0.67	3.53	4.48	3.57	2.68	0.63	0.63	1.05	1.19	0.97	1.30	3.85	3.40	2.78	2.66
**2C8L**	3.70	3.70	2.75	0.58	0.34	0.32	0.00	4.34	3.97	2.92	3.68	2.99	2.91	0.99	2.87	0.93	0.67	3.54	4.41	3.56	2.71	0.65	0.62	1.04	1.11	0.95	1.31	3.72	3.43	3.03	2.70
**2C9M**	2.14	2.13	3.43	4.47	4.24	4.39	4.34	0.00	3.11	3.57	2.13	2.84	2.95	4.19	3.15	4.20	4.37	4.18	2.57	2.14	3.60	4.51	4.74	4.23	4.09	4.21	4.24	2.26	2.10	3.09	3.07
**3B9B**	3.89	3.82	3.50	3.73	3.92	3.78	4.01	3.11	0.00	3.09	3.82	3.11	3.17	3.50	3.15	3.53	3.75	4.12	2.53	3.83	3.09	4.00	3.80	3.77	3.40	3.61	3.35	3.79	1.79	1.82	3.50
**3B9R**	4.65	4.57	1.54	2.79	2.82	2.83	2.92	3.57	3.11	0.00	4.65	1.08	1.09	2.87	1.09	2.93	2.78	3.20	2.41	4.49	1.08	3.00	2.94	2.90	2.92	2.88	3.26	4.51	2.59	2.26	1.42
**3BA6**	0.46	0.43	4.53	3.61	3.42	3.57	3.61	2.16	3.82	4.65	0.00	4.39	4.62	3.67	4.75	3.52	3.79	4.44	2.23	0.87	4.64	3.60	3.53	3.46	3.43	3.44	3.90	0.74	4.33	4.29	4.81
**3FGO**	4.50	4.37	1.50	2.99	2.94	2.99	3.01	2.84	3.15	1.09	4.39	0.00	1.00	2.94	0.99	3.01	2.92	2.97	2.42	4.43	1.08	3.14	3.09	3.15	3.13	2.97	3.24	4.54	2.63	2.19	1.44
**3FPB**	4.89	4.78	1.34	3.00	2.90	2.88	2.92	2.95	3.17	1.09	4.62	1.00	0.00	2.92	0.34	3.01	2.82	2.86	2.45	4.63	0.92	3.02	2.99	3.09	3.13	2.95	3.21	4.83	2.62	2.37	1.15
**3FPS**	3.84	3.62	2.86	1.03	0.99	1.00	0.99	4.19	3.48	2.87	3.68	2.94	2.93	0.00	2.92	0.19	1.01	3.58	4.17	3.73	2.90	0.97	0.94	0.92	0.86	0.85	1.10	4.02	3.00	2.57	2.78
**2O9J**	4.89	4.97	1.34	2.94	2.84	2.96	2.92	3.15	3.13	1.09	4.73	0.99	0.34	2.91	0.00	3.00	2.94	2.91	2.51	4.72	0.84	3.02	2.98	3.10	3.07	2.98	3.23	4.79	2.58	2.38	1.08
**2OA0**	3.81	3.50	2.86	0.97	0.93	0.93	0.93	4.21	3.56	2.89	3.37	3.01	3.01	0.19	2.99	0.00	0.98	3.54	4.03	3.56	2.79	0.88	0.88	0.91	0.89	0.84	1.12	3.88	2.95	2.66	2.78
**1IWO**	3.81	3.73	2.72	0.81	0.68	0.67	0.67	4.40	3.80	2.77	3.79	2.92	2.84	1.01	2.90	0.97	0.00	3.48	4.04	3.68	2.69	0.62	0.64	1.04	1.11	0.99	1.30	3.90	3.36	3.04	2.66
**1KJU**	4.23	4.45	2.80	3.56	3.53	3.53	3.49	4.14	4.08	3.19	4.36	3.02	2.86	3.50	2.91	3.52	3.48	0.00	2.97	4.25	2.82	3.46	3.59	3.59	3.56	3.53	3.50	4.43	3.75	3.68	2.81
**1SU4**	2.30	2.28	2.39	4.51	4.83	4.45	4.50	2.57	2.53	2.42	2.23	2.47	2.42	4.17	2.44	4.00	4.10	2.97	0.00	2.29	2.55	4.60	4.75	3.80	3.77	3.91	4.48	2.40	3.69	1.92	2.44
**1VFP**	0.84	0.86	4.45	3.68	3.63	3.60	3.60	2.14	3.84	4.52	0.87	4.45	4.63	3.74	4.72	3.56	3.69	4.25	2.29	0.00	4.50	3.71	3.85	3.44	3.27	3.68	3.84	0.70	4.20	4.13	4.58
**1WPG**	4.60	4.65	1.10	2.69	2.73	2.67	2.72	3.59	3.08	1.07	4.64	1.09	0.92	2.85	0.84	2.79	2.63	2.82	2.55	4.46	0.00	2.83	2.78	2.91	2.93	2.77	2.98	4.62	2.47	2.18	0.96
**2AGV**	3.75	3.63	2.68	0.83	0.72	0.63	0.65	4.51	3.85	3.00	3.60	3.13	3.01	0.90	3.04	0.88	0.62	3.43	4.60	3.73	2.82	0.00	0.38	0.96	1.09	0.94	1.25	3.77	3.59	3.02	2.80
**2DQS**	3.80	3.62	2.71	0.78	0.67	0.63	0.62	4.80	3.85	2.90	3.59	3.09	2.99	0.94	2.98	0.88	0.64	3.59	4.74	3.80	2.78	0.38	0.00	0.98	1.16	0.94	1.22	3.89	3.49	3.15	2.70
**2EAR**	3.56	3.49	2.75	1.02	0.99	1.05	1.04	4.19	3.77	2.93	3.46	3.17	3.10	0.92	3.12	0.90	1.04	3.59	3.87	3.44	2.92	0.96	0.97	0.00	0.86	0.78	1.44	3.59	3.56	2.71	2.83
**2EAS**	3.36	3.28	2.90	1.18	1.11	1.19	1.10	4.09	3.37	2.95	3.55	3.18	3.12	0.86	3.10	0.89	1.11	3.62	3.74	3.26	2.94	1.09	1.16	0.86	0.00	0.82	1.23	3.43	3.29	3.02	2.88
**2EAT**	3.42	3.45	2.70	1.05	0.95	0.97	0.95	4.16	3.61	2.88	3.51	2.98	2.88	0.86	2.94	0.84	0.98	3.53	3.91	3.62	2.77	0.94	0.94	0.78	0.82	0.00	1.44	3.43	3.38	2.94	2.76
**2EAU**	3.86	3.86	2.83	1.39	1.33	1.30	1.31	4.24	3.35	3.23	3.81	3.24	3.21	1.11	3.27	1.13	1.30	3.50	4.48	3.80	2.98	1.25	1.22	1.45	1.24	1.44	0.00	4.04	3.39	3.18	3.16
**2ZBD**	0.71	0.71	4.60	3.78	3.63	3.85	3.73	2.24	3.75	4.47	0.74	4.48	4.69	3.98	4.77	3.88	3.85	4.40	2.37	0.70	4.62	3.79	3.89	3.57	3.46	3.45	3.99	0.00	4.36	4.25	4.73
**2ZBE**	4.18	4.15	2.83	3.51	3.44	3.40	3.41	2.05	1.79	2.59	4.33	2.64	2.62	2.98	2.57	3.04	3.35	3.74	3.69	4.17	2.47	3.51	3.49	3.55	3.25	3.52	3.41	4.41	0.00	1.58	2.74
**2ZBF**	4.25	4.28	2.60	2.83	2.72	2.70	3.02	3.09	1.83	2.24	4.28	2.21	2.36	2.58	2.38	2.53	3.04	3.68	1.92	4.11	2.18	3.04	3.14	2.70	3.00	2.94	3.16	4.33	1.58	0.00	2.42
**2ZBG**	4.88	4.85	0.97	2.66	2.74	2.68	2.68	3.07	3.46	1.43	4.76	1.44	1.15	2.78	1.08	2.78	2.67	2.81	2.44	4.58	0.96	2.80	2.70	2.83	2.88	2.74	3.17	4.75	2.74	2.45	0.00

Supplementary Table S4. Pruned C-alpha r.m.s. deviation between the initially identified Ca2+-ATPase scaffolds. Models with a deviation less than 1 Å were removed from Supplementary Table S3. The resulting list shown here was used for the initial 14 scaffolds to generate the search library. The code refers to the scaffolds pdb-id.

	1T5S	1XP5	2BY4	2C9M	3B9B	3B9R	3FGO	3FPB	3FPS	1KJU	1SU4	2EAU	2ZBE	2ZBF
**1T5S**	0.00	4.56	3.63	2.14	3.90	4.74	4.53	4.88	3.85	4.25	2.29	3.84	4.27	4.31
**1XP5**	4.55	0.00	2.65	3.44	3.48	1.54	1.50	1.34	2.84	2.79	2.39	2.83	2.83	2.64
**2BY4**	3.63	2.67	0.00	4.36	3.83	2.83	2.92	3.00	1.03	3.56	4.51	1.38	3.48	2.85
**2C9M**	2.14	3.43	4.47	0.00	3.11	3.57	2.84	2.95	4.19	4.18	2.57	4.24	2.10	3.09
**3B9B**	3.89	3.50	3.73	3.11	0.00	3.09	3.11	3.17	3.50	4.12	2.53	3.35	1.79	1.82
**3B9R**	4.65	1.54	2.79	3.57	3.11	0.00	1.08	1.09	2.87	3.20	2.41	3.26	2.59	2.26
**3FGO**	4.50	1.50	2.99	2.84	3.15	1.09	0.00	1.00	2.94	2.97	2.42	3.24	2.63	2.19
**3FPB**	4.89	1.34	3.00	2.95	3.17	1.09	1.00	0.00	2.92	2.86	2.45	3.21	2.62	2.37
**3FPS**	3.84	2.86	1.03	4.19	3.48	2.87	2.94	2.93	0.00	3.58	4.17	1.10	3.00	2.57
**1KJU**	4.23	2.80	3.56	4.14	4.08	3.19	3.02	2.86	3.50	0.00	2.97	3.50	3.75	3.68
**1SU4**	2.30	2.39	4.51	2.57	2.53	2.42	2.47	2.42	4.17	2.97	0.00	4.48	3.69	1.92
**2EAU**	3.86	2.83	1.39	4.24	3.35	3.23	3.24	3.21	1.11	3.50	4.48	0.00	3.39	3.18
**2ZBE**	4.18	2.83	3.51	2.05	1.79	2.59	2.64	2.62	2.98	3.74	3.69	3.41	0.00	1.58
**2ZBF**	4.25	2.60	2.83	3.09	1.83	2.24	2.21	2.36	2.58	3.68	1.92	3.16	1.58	0.00

Supplementary Table S5. Models identified by BLAST, used to generate the domains for the search model library.

**Domain**	**pdb**	**CopA coverage (%)**	**seq. id to CopA (%)**	**C(alpha) r.m.s.d. to final CopA model**
A	2hc8	102 / 732 (13.9%)	57 / 102 (55.9%)	1.06 Å
N	3a1c	119 / 732 (16.3%)	44 / 119 (37.0%)	1.55 Å
P	3a1c	146 / 732 (19.9%)	75 / 146 (51.4%)	1.48 Å
M16 (Ca^{2+}-ATPase)	14 different[a]	152 / 732 (20.7%)	41 / 152 (27.0%)	2.58 Å - 4.12 Å
M16 (H^{+}-ATPase)	3b8c	185 / 732 (25.3%)	38 / 185 (20.5%)	3.06 Å
Ca^{2+}-ATPase (P+N+A+M16)	-	519 / 732 (70.9%)	217 / 519 (41.8%)	-
H^{+}-ATPase (P+N+A+M16)	-	552 / 732 (75.4%)	214 / 552 (38.8%)	-

a) For the M16 domain, the relevant part of the individual scaffolds were used, since M16 displays considerable intra-domain variation (cf. Supplementary Table S4 for a list of these scaffolds).

Supplementary Table S6. r.m.s.d. of search-models with no truncation, i.e. all domains (A+N+P+M16). Pdbs are numbered 1-15. Their order is identical to the order seen in Figure 2C and Supplementary Table S4, with the addition of the H+-ATPase (3b8c) added as a scaffold (pdb15). Red notes r.m.s.d. below 1 Å.

	pdb1	pdb2	pdb3	pdb4	pdb5	pdb6	pdb7	pdb8	pdb9	pdb10	pdb11	pdb12	pdb13	pdb14	pdb15
**pdb1**	0.00	4.24	3.67	2.68	3.93	4.09	3.98	4.23	3.80	4.43	2.29	3.69	3.67	3.92	3.04
**pdb2**	4.24	0.00	2.82	2.11	3.15	1.37	1.41	1.91	2.94	2.84	2.09	3.05	2.70	2.47	3.91
**pdb3**	3.67	2.82	0.00	5.15	3.97	2.88	3.14	3.02	0.94	3.26	4.12	1.13	3.77	3.04	3.66
**pdb4**	2.68	2.11	5.15	0.00	1.97	2.15	2.88	2.93	3.72	3.02	2.82	5.16	2.84	2.14	3.43
**pdb5**	3.92	3.15	3.98	1.97	0.00	2.75	2.91	3.34	3.97	4.07	2.11	4.10	1.39	1.73	4.40
**pdb6**	4.09	1.37	2.87	2.15	2.74	0.00	0.92	1.57	3.12	3.23	2.55	3.20	2.39	1.87	4.74
**pdb7**	4.17	1.41	3.14	2.88	2.94	0.92	0.00	1.41	3.27	3.21	2.35	3.35	2.53	2.07	3.74
**pdb8**	4.23	1.91	3.26	2.93	3.31	1.63	1.41	0.00	3.23	3.52	3.20	3.35	2.98	2.62	3.85
**pdb9**	3.83	2.94	0.94	3.72	3.97	3.12	3.08	3.23	0.00	3.27	4.03	0.94	3.71	3.29	3.64
**pdb10**	4.43	2.83	3.26	3.02	4.00	3.23	3.21	3.52	3.27	0.00	2.87	3.25	3.63	3.31	4.89
**pdb11**	2.29	2.09	4.12	2.82	2.11	2.55	2.35	3.18	4.03	2.87	0.00	6.44	1.76	4.66	2.53
**pdb12**	3.82	3.05	1.13	5.16	4.10	3.05	3.39	3.32	0.94	3.25	6.42	0.00	3.99	3.38	3.54
**pdb13**	3.67	2.70	3.77	2.84	1.39	2.39	2.53	2.98	3.98	3.62	1.76	3.99	0.00	1.71	4.30
**pdb14**	3.83	2.47	3.06	2.13	1.73	1.87	2.08	2.62	3.29	3.31	4.52	3.31	1.65	0.00	4.58
**pdb15**	3.02	3.88	3.68	3.56	4.41	4.74	3.86	3.86	3.64	4.74	2.51	3.55	4.04	4.09	0.00

Supplementary Table S7. r.m.s.d. of search-models with no N domain (A+P+TM16). Pdbs are numbered 1-15. Their order is identical to the order seen in Figure 2C and Supplementary Table 4, with the addition of the H+-ATPase (3b8c) added as a scaffold (pdb15). Red notes r.m.s.d. below 1 Å.

	pdb1	pdb2	pdb3	pdb4	pdb5	pdb6	pdb7	pdb8	pdb9	pdb10	pdb11	pdb12	pdb13	pdb14	pdb15
**pdb1**	0.00	4.14	3.39	2.67	3.96	4.01	3.70	4.13	3.61	3.99	2.27	3.46	4.08	3.83	2.51
**pdb2**	4.14	0.00	2.39	3.97	2.75	1.12	1.08	1.17	2.44	2.58	4.57	2.52	2.67	1.76	3.22
**pdb3**	3.44	2.39	0.00	3.76	3.53	2.08	2.25	2.37	0.89	3.82	3.76	1.11	3.15	2.52	2.59
**pdb4**	2.67	3.96	3.76	0.00	3.77	3.87	3.79	4.11	3.70	4.42	3.38	3.73	3.99	4.03	3.22
**pdb5**	3.96	2.79	3.53	3.95	0.00	2.64	2.79	2.68	3.52	3.47	3.82	3.49	1.37	1.82	3.70
**pdb6**	4.01	1.12	2.08	3.94	2.55	0.00	0.88	0.97	2.13	2.89	4.29	2.16	2.56	1.68	3.97
**pdb7**	3.86	1.08	2.25	3.79	2.80	0.88	0.00	0.76	2.22	2.65	4.38	2.26	2.69	1.77	3.11
**pdb8**	4.26	1.17	2.37	4.08	2.68	0.97	0.76	0.00	2.32	2.87	4.40	2.32	2.62	1.94	3.30
**pdb9**	3.66	2.44	0.91	3.69	3.52	2.13	2.22	2.32	0.00	3.89	3.48	1.01	3.15	2.65	2.51
**pdb10**	3.99	2.56	3.82	4.42	3.47	2.89	2.65	2.87	3.89	0.00	4.85	3.77	3.27	2.48	3.82
**pdb11**	2.27	4.56	3.76	3.38	3.82	4.29	4.17	4.36	3.48	4.84	0.00	3.50	4.17	4.17	2.52
**pdb12**	3.56	2.52	1.11	3.73	3.49	2.16	2.26	2.32	1.01	3.77	3.50	0.00	3.30	2.67	2.51
**pdb13**	3.80	2.67	3.15	3.99	1.38	2.58	2.69	2.64	3.15	3.27	4.27	3.30	0.00	1.76	3.79
**pdb14**	3.84	1.76	2.52	4.03	1.82	1.68	1.77	1.94	2.65	2.48	4.22	2.67	1.76	0.00	3.82
**pdb15**	2.43	3.24	2.58	3.60	3.78	4.07	3.07	3.62	2.42	3.71	2.48	2.51	3.53	3.28	0.00

Supplementary Table S8. r.m.s.d. of search-models with no A domain (N+P+TM16). Pdbs are numbered 1-15. Their order is identical to the order seen in Figure 2C and Supplementary Table 4, with the addition of the H+-ATPase (3b8c) added as a scaffold (pdb15). Red notes r.m.s.d. below 1 Å.

	pdb1	pdb2	pdb3	pdb4	pdb5	pdb6	pdb7	pdb8	pdb9	pdb10	pdb11	pdb12	pdb13	pdb14	pdb15
**pdb1**	0.00	4.12	3.58	1.94	3.51	3.75	3.80	4.14	3.72	4.31	1.82	3.60	3.64	3.88	2.96
**pdb2**	4.09	0.00	2.13	2.11	3.07	1.48	1.44	2.06	2.33	2.57	2.09	2.29	2.43	2.36	3.80
**pdb3**	3.57	2.13	0.00	5.13	3.68	2.40	2.50	2.56	1.02	2.62	5.83	1.03	3.39	2.65	3.38
**pdb4**	1.94	2.11	5.13	0.00	1.97	2.15	2.18	2.93	4.40	3.02	1.24	5.12	2.84	2.95	2.25
**pdb5**	3.52	3.07	3.61	1.97	0.00	2.56	2.81	3.56	3.63	3.92	2.11	3.94	1.36	1.91	4.12
**pdb6**	3.78	1.48	2.40	2.15	2.57	0.00	0.90	1.85	2.59	3.12	2.55	2.42	2.14	1.67	3.72
**pdb7**	3.80	1.44	2.45	2.18	2.80	0.90	0.00	1.52	2.67	3.16	2.22	2.48	2.31	1.84	3.69
**pdb8**	4.14	2.02	2.56	2.93	3.56	1.85	1.52	0.00	2.69	3.37	3.07	2.50	2.83	2.57	3.78
**pdb9**	3.72	2.33	1.02	4.40	3.76	2.59	2.67	2.69	0.00	2.74	5.20	0.86	3.27	2.76	3.41
**pdb10**	4.50	2.57	2.62	3.02	3.94	3.12	3.16	3.37	2.74	0.00	2.87	2.71	3.51	3.35	4.79
**pdb11**	1.82	2.09	5.79	1.24	2.11	2.55	2.22	3.07	5.20	2.87	0.00	6.45	2.34	4.10	2.33
**pdb12**	3.64	2.29	1.03	5.11	3.97	2.42	2.49	2.50	0.86	2.70	6.45	0.00	3.52	2.78	3.34
**pdb13**	3.61	2.43	3.39	2.84	1.36	2.14	2.39	2.83	3.28	3.51	2.34	3.52	0.00	1.72	4.00
**pdb14**	3.79	2.36	2.65	2.95	1.91	1.67	1.84	2.57	2.76	3.34	4.10	2.78	1.73	0.00	3.64
**pdb15**	2.96	3.69	3.47	2.23	4.14	3.72	3.67	3.78	3.37	4.78	2.35	3.35	4.06	3.66	0.00

Supplementary Table S9. r.m.s.d. of search-models with no TM16 domain (A+N+P). Pdbs are numbered 1-15. Their order is identical to the order seen in Figure 2C and Supplementary Table S4, with the addition of the H+-ATPase (3b8c) added as a scaffold (pdb15). Red notes r.m.s.d. below 1 Å. Note that without the transmembrane domain present the rmsd drops in may cases (compare Supplementary Table S9 to Supplementary Tables S6-S8). Especially pdb2, pdb6 and pdb7 are similar, and pdb3, pdb9 and pdb12 are similar as expected from the conformations that they represent.

	pdb1	pdb2	pdb3	pdb4	pdb5	pdb6	pdb7	pdb8	pdb9	pdb10	pdb11	pdb12	pdb13	pdb14	pdb15
**pdb1**	0.00	2.01	3.40	0.97	1.66	2.02	1.96	3.01	2.96	4.07	2.86	3.16	2.46	1.73	2.97
**pdb2**	3.54	0.00	3.17	2.09	2.01	0.58	0.56	1.66	3.50	2.66	2.01	3.31	2.07	1.98	4.18
**pdb3**	3.40	3.17	0.00	3.90	4.19	3.38	3.52	3.66	0.59	2.98	3.88	0.53	3.85	4.25	3.62
**pdb4**	0.90	2.09	3.90	0.00	1.21	2.10	2.11	2.95	4.21	2.90	2.82	4.06	1.37	1.61	3.38
**pdb5**	3.53	1.99	4.00	1.21	0.00	1.90	2.02	2.90	4.54	2.57	1.24	4.32	0.75	0.59	4.10
**pdb6**	3.12	0.58	3.38	2.10	1.90	0.00	0.54	1.68	3.51	2.68	2.00	3.37	1.94	1.71	3.56
**pdb7**	3.09	0.56	3.52	2.11	2.02	0.54	0.00	1.40	3.73	2.66	2.15	3.59	2.06	1.82	3.36
**pdb8**	3.01	1.65	3.66	2.93	2.90	1.68	1.40	0.00	3.83	3.07	3.03	3.53	2.91	2.72	3.91
**pdb9**	3.03	3.50	0.59	4.21	4.51	3.51	3.73	3.83	0.00	3.23	4.23	0.45	4.03	4.57	3.39
**pdb10**	4.56	2.65	2.98	2.90	2.53	2.68	2.66	3.07	3.11	0.00	2.79	2.92	2.64	2.54	4.57
**pdb11**	2.86	2.01	3.88	2.82	1.24	2.00	2.15	3.03	4.23	2.79	0.00	4.06	1.63	1.73	2.02
**pdb12**	3.17	3.31	0.53	4.06	4.32	3.37	3.59	3.20	0.45	3.20	4.06	0.00	4.02	4.28	3.44
**pdb13**	2.46	2.07	3.85	1.37	0.75	1.94	2.06	2.91	4.03	2.64	1.63	4.09	0.00	0.83	4.46
**pdb14**	2.60	1.98	4.25	1.61	0.59	1.71	1.81	2.72	4.57	2.54	1.73	4.28	0.83	0.00	3.93
**pdb15**	2.97	4.18	3.63	3.32	4.16	3.56	3.36	3.91	3.39	4.57	2.02	3.46	4.46	3.85	0.00

Supplementary scripts

Contains 6 example scripts:

`setup_search.sh`

`start_runs_setup.sh`

`eval_result.sh`

`create_searchmodel_variations.sh`

`phaser_and_analysis_for_setup.sh`

`evaluate_for_setup.sh`

Brief guide:

The scripts are hopefully relatively self-explanatory. The example scripts here show 3 datasets being tested with 4 scaffolds, each having 2 model variations (all atoms and poly-alanine), using 2 resolution-limits and 2 r.m.s.d. values on a 12 cpu-core cluster (a total of 240 runs). The scripts assumes the directory structure and file-placement mentioned below which should be created manually. Follow the steps noted here to initiate the MRPM search:

1) mkdir $MRPM (it is the root directory and can have any name).

2) mkdir $MRPM/models.

3) mkdir $MRPM/input.

4) Place 'setup_search.sh', 'start_runs_setup.sh', 'eval_result.sh' in $MRPM.

5) Place 'phaser_and_analysis_for_setup.sh' and 'evaluate_for_setup.sh' in $MRPM/input.

6) Place 'create_searchmodel_variations.sh' in $MRPM/models.

7) Place 'target.fas' (containing the target-sequence in FASTA) in $MRPM/input.

8) Place all datasets to test in $MRPM/input and name then data1.mtz, data2.mtz etc.
Datasets should contain the following columns: H,K,L,FP,SIGFP.

10) Place the HA dataset to search for anomalous peaks in $MRPM/input and name ha-data.mtz.
HA Dataset should contain the following columns: H,K,L,DANO,SIGDANO.

11) In $MRPM/models, create subdirectories called scaffold1, scaffold2 etc. One for each scaffold to test. Copy the pdb's to use as domains and scaffold into each scaffold-subdirectory.

12) Using pymol or similar, overlay the domains to the scaffold and save the final result as searchmodel.pdb.

12) Edit and run $MRPM/models/create_searchmodel.sh to generate the search-model library.

13) Edit $MRPM/input/phaser_and_analysis_for_setup.sh to set up the parameters to scan in the individual MR runs.

14) Edit $MRPM/input/evaluate_for_setup.sh to set up the parameters used to calculate the anomalous difference maps.

15) Edit and run $MRPM/setup_search.sh to set up the directory structure and input files for the search.

16) Edit and run $MRPM/start_runs_setup.sh to set up a 'start_runs.sh' file that will initiate MRPM on a given number of cores.

17) Run $MRPM/start_runs.sh to initiate MRPM.

18) During and after the runs have finished run $MRPM/eval_result.sh to list the results from individual runs that have completed. Use GNUPLOT or similar to plot the results if desired.

Script S1: setup_search.sh

```
#!/bin/sh
#######################
#
# file 'setup_search.sh'
# setup the final data-structure and input files before initiating MRPM
# by Bjørn Panyella Pedersen,
# PUMPKIN centre, Aarhus University
#
#######################

dataarray="1 2 3"
scaffoldarray="1 2 3 4"
modelarray="1 2"

for d in ${dataarray}
do
    for s in ${scaffoldarray}
    do
        for m in ${modelarray}
        do
            # set up directory structure
            if [ ! -d ./data${d} ]; then mkdir data${d};fi
            cd data${d}
            if [ ! -d ./scaffold${s} ]; then mkdir scaffold${s};fi
            cd scaffold${s}
            if [ ! -d ./model${m} ]; then mkdir model${m};fi
            cd model${m}
            if [ ! -d ./output ]; then mkdir output;fi
            # now get the script
            cp ../../../input/phaser_and_analysis_for_setup.sh ./phaser_and_analysis.sh
            # set the dataset
            sed -i -e "s/<data>/data${d}\.mtz/" ./phaser_and_analysis.sh
            # set the scaffold
            sed -i -e "s/<scaffold>/scaffold${s}/" ./phaser_and_analysis.sh
            # set the model
            sed -i -e "s/<model>/model${m}\.pdb/" ./phaser_and_analysis.sh
            # Get the HA-peak evaluation script
            cp ../../../input/evaluate_for_setup.sh ./evaluate.sh
            # return to start
            cd ../../..
        done
    done
done
echo ""
echo " All done..."
echo ""
```

Script S2: start_runs_setup.sh

```
#! /bin/sh
#######################
#
# file 'start_runs_setup.sh'
# setup to run multiple MRPM jobs on multiple cpus
# by Bjørn Panyella Pedersen,
# PUMPKIN centre, Aarhus University
#
#######################

noofcpu="12"
dataarray="1 2 3"
scaffoldarray="1 2 3 4"
modelarray="1 2"

echo ""
echo " Remember to edit this file to fit the experiment."
echo ""
echo " ___input___"
echo " noofcpu: $noofcpu"
echo " dataarray: $dataarray"
echo " scaffoldarray: $scaffoldarray"
echo " modelarray: $modelarray"

#cleanup
touch tmp.runlist start_runs.sh .cpu_tmp
rm tmp.runlist start_runs.sh .cpu_*

for d in ${dataarray}
do
    for s in ${scaffoldarray}
    do
        for m in ${modelarray}
        do
            echo "cd ./data${d}/scaffold${s}/model${m};sh phaser_and_analysis.sh;cd ../../.." >>tmp.runlist
        done
```

```
    done
done

# split up the runs
split -l $(echo $(cat tmp.runlist | wc -l)/${noofcpu} +1| bc) tmp.runlist .cpu_

for file in `echo ./.cpu_*`
do
echo "nohup sh $file >${file}_log &" >>start_runs.sh
done

#cleanup
rm tmp.runlist

echo ""
echo " run start_runs.sh to execute the search"
echo " All done..."
echo ""
```

Script S3: eval_result.sh

```
#! /bin/sh
#######################
#
# file 'eval_result.sh'
# Evaluate MRPM
# by Bjørn Panyella Pedersen,
# PUMPKIN centre, Aarhus University
#
#######################
if [ ! $# = "1" ]; then
  echo ""
  echo " usage 'eval_result.sh <sort-keyword>'"
  echo " options: none name llg z ha"
  echo ""
  echo " use none for a quick view since no sort-argument is called"
  echo "only 'data*' directories are searched for solutions"
  echo ""
  exit
fi
# set key
key=$1

# get total number of runs
ta=`grep "array=" input/phaser_and_analysis_for_setup.sh | awk '{printf("*%s", NF)}' | xargs echo "1"|bc`
tb=`grep "array=" setup.sh | awk '{printf("*%s", NF)}' | xargs echo "1"|bc`
total=`echo "$ta*$tb"|bc`

# run the find command
if [ "${key}" = "none" ]; then
  find ./data* -name \*.summary | xargs cat >junktmp
  sol=`cat junktmp | wc -l`
  fail=`grep " -" junktmp | wc -l`
  part=`grep " yes" junktmp | wc -l`
  true=`echo "$sol-$fail-$part" |bc`
  cat junktmp
  rm junktmp
  echo " $sol/${total} runs completed ($true solutions, $part partial solutions and $fail with no solution)"
elif [ "${key}" = "name" ]; then
  find ./data* -name \*.summary | xargs cat >junktmp
  sort -n -k1 junktmp >junktmp2
  sol=`cat junktmp2 | wc -l`
  fail=`grep " -" junktmp | wc -l`
  part=`grep " yes" junktmp | wc -l`
  true=`echo "$sol-$fail-$part" |bc`
  cat junktmp2
  rm junktmp junktmp2
  echo " $sol/${total} runs completed ($true solutions, $part partial solutions and $fail with no solution)"
elif [ "${key}" = "llg" ]; then
  find ./data* -name \*.summary | xargs cat >junktmp
  sort -n -k14 junktmp >junktmp2
  sol=`cat junktmp2 | wc -l`
  fail=`grep " -" junktmp | wc -l`
  part=`grep " yes" junktmp | wc -l`
  true=`echo "$sol-$fail-$part" |bc`
  cat junktmp2
  rm junktmp junktmp2
  echo " $sol/${total} runs completed ($true solutions, $part partial solutions and $fail with no solution)"
elif [ "${key}" = "z" ]; then
  find ./data* -name \*.summary | xargs cat >junktmp
  sort -n -k16 junktmp >junktmp2
  sol=`cat junktmp2 | wc -l`
  fail=`grep " -" junktmp | wc -l`
  part=`grep " yes" junktmp | wc -l`
  true=`echo "$sol-$fail-$part" |bc`
  cat junktmp2
  rm junktmp junktmp2
```

```
  echo " $sol/${total} runs completed ($true solutions, $part partial solutions and $fail with no solution)"
  elif [ "${key}" = "ha" ]; then
  find ./data* -name \*.summary | xargs cat >junktmp
  sort -n -k18 junktmp >junktmp2
  sol=`cat junktmp2 | wc -l`
  fail=`grep " -" junktmp | wc -l`
  part=`grep " yes" junktmp | wc -l`
  true=`echo "$sol-$fail-$part" |bc`
  cat junktmp2
  rm junktmp junktmp2
  echo " $sol/${total} runs completed ($true solutions, $part partial solutions and $fail with no solution)"
else
  echo " sort-keyword unknown"
  echo " options: none name llg z ha"
fi
```

Script S4: create_searchmodel_variations.sh

```
#!/bin/sh
######################
#
# file 'create_searchmodel_variations.sh'
# merge and renumber scaffold input to generate models for MRPM
# by Bjørn Panyella Pedersen,
# PUMPKIN centre, Aarhus University
#
# model1: full model
# model2: full model, polyalanine
#
######################

# loop though the following scaffolds:
i="1"
max="4"
while [ ${i} -le ${max} ]
do

# test that the scaffold dir exist
if [ ! -d ./scaffold${i} ]; then echo " FATAL ERROR: ./scaffold${i}/ is missing";exit; fi

cd ./scaffold${i}

# test for needed input
if [ ! -e searchmodel.pdb ]; then echo " FATAL ERROR: ./scaffold${i}/searchmodel.pdb is missing";exit; fi

# cleanup the input pdb
#remove AnisoU records from searchmodel and keep only chain A
pdbcur xyzin searchmodel.pdb xyzout searchmodel_tmp1.pdb <<EOF
lvchain A
noanisou
delhydrogen
delsolvent
end
EOF

# further cleanup
grep -v "CONECT" searchmodel_tmp1.pdb >searchmodel_tmp2.pdb
grep -v "MASTER" searchmodel_tmp2.pdb >searchmodel_tmp1.pdb

#reset bfactor
pdbset xyzin searchmodel_tmp1.pdb xyzout searchmodel_tmp2.pdb <<EOF
bfactor 50
end
EOF

# Model1, no changes
cp searchmodel_tmp2.pdb model1.pdb

# Model2, polyalanine version
pdbset XYZIN model1.pdb XYZOUT model2.pdb <<EOF
excl sidech
end
EOF

##
#
# insert additional search-model variations here as needed
#
##

# final cleanup
rm *tmp*

cd ..
echo ""
echo "  scaffold${i} done..."
echo ""
```

```
i=`echo "${i} + 1" | bc`
done

echo ""
echo "  All done..."
```

Script S5: phaser_and_analysis_for_setup.sh

```
#! /bin/sh
#######################
#
# file 'phaser_and_analysis_for_setup.sh'
# input file to run the actual phaser-job (setup for phenix.phaser v2.3)
# by Bjørn Panyella Pedersen,
# PUMPKIN centre, Aarhus University
#
#######################

resarray="0.0 6.0 8.0"
rmsdarray="2 3"
pack="30"
number="1"
topfiles="10"
data="../../../input/<data>"
seq="../../../input/target.fas"
model="../../../models/<scaffold>/<model>"

# get dataid and pdbid for the final output.
dataid=`pwd | awk -F "/" '{printf("%s", $(NF-2))}'`
scaffoldid=`pwd | awk -F"/" '{printf("%s", $(NF-1))}'`
modelid=`pwd | awk -F"/" '{printf("%s", $NF)}'`
run="1"

for res in ${resarray}
do
    for rmsd in ${rmsdarray}
    do
        if [ ! -e ./result_run${run}.summary ]; then phenix.phaser << EOF > phaser_run${run}.log; fi
MODE MR_AUTO
HKLIN $data
LABIN  F=FP SIGF=SIGFP
TITLE find target using ${model}.pdb
COMPOSITION PROTEIN SEQ $seq &
    NUMBER $number
RESOLUTION 100.0 $res
ENSEMBLE $model &
    PDBFILE ${model}.pdb &
    RMS $rmsd
SEARCH ENSEMBLE $model NUMBER $number
PACK CUTOFF $pack
PACK SELECT ALLOW
FINAL ROT SELECT PERCENT 75.0
FINAL TRA SELECT PERCENT 75.0
SAVE ROT CLUSTER ON DUMP 20
SAVE TRA CLUSTER ON DUMP 20
PERMUTATIONS OFF
ROOT ./output/phaser_run${run}
HKLOUT ON
TOPFILES $topfiles
EOF

        # now calculate the HA-anom map..
        if [ ! -e ./result_run${run}.summary ]; then evaluate.sh ${run}; fi

        # now output the result in a nice simple way
        llg="-"
        z="-"
        ha="-"
        sol="-"
        z2="-"
        if [ -e output/phaser_run${run}.1.pdb ]; then llg=`awk 'NR==3 {printf("%s", $(NF-1))}' output/phaser_run$
{run}.1.pdb | cut -c5-`; fi
        if [ -e output/phaser_run${run}.1.pdb ]; then z=`awk 'NR==3 {printf("%s", $NF)}' output/phaser_run$
{run}.1.pdb | cut -c5-`; fi
        if [ -e result_run${run}.txt ]; then ha=`awk 'NR==2 {printf("%s", $2)}' result_run${run}.txt `; fi
        if [ -e result_run${run}.txt ]; then sol=`awk 'NR==2 {printf("%s", $1)}' result_run${run}.txt | cut -d"-"
-f2 | bc`; fi
        if [ -e result_run${run}.txt ]; then z2=`awk 'NR==3 {printf("%s", $3)}' output/phaser_run${run}.${sol}.pdb
| cut -c5-`; fi
        printf "data: %-6s  scaffold: %-10s  model: %6s  run: %2s  res: %3s  rmsd: %1s  LLG: %4s  Z: %4s  HA: %4s
from_sol: %2s with_z: %4s\n" $dataid $scaffoldid $modelid $run $res $rmsd $llg $z $ha $sol $z2 >result_run$
{run}.summary
        run=`echo "${run} + 1" | bc`
    done
done
```

Script S6: evaluate_for_setup.sh

```
#! /bin/sh
######################
#
# file 'evaluate_for_setup.sh'
# file to run the HA anomalous map calculations
# by Bjørn Panyella Pedersen,
# PUMPKIN centre, Aarhus University
#
######################

if [ $# = "0" ]; then
  echo ""
  echo "ERROR"
  echo ""
  echo "usage 'evaluate.sh <phaserrun-number to test> [purge]'"
  echo "purge keywork is optional and will force rewrite of all data"
  echo "output is the top anomalous peak from each phaser solution"
  echo ""
  exit
fi

# HA data to test against
data="../../../input/ha-data.mtz"

# fft ano map cutoff to test (3 currently allowed)
cutoff1="9"
cutoff2="7.5"
cutoff3="6"

# number of max solutions from phaser to test
maxsoltotest="10"

# set run number
run=$1

# ugly fix
touch ./output/phaser_run${run}.1.mtz

# start analysis
for model in `echo ./output/phaser_run${run}.*.mtz`
do

# exit if there was no solution
if [ ! -s ./output/phaser_run${run}.1.mtz ];then rm ./output/phaser_run${run}.1.mtz; exit; fi

# get pdb name
pdb=`basename "$model" .mtz`
# get solution number
sol=`basename "$model" | cut -d "." -f2`
# get FOM
fom=`mtzdmp $model | grep "FOM" | tail -n1 | awk '{printf($7)}'`

# cad phaser-phases to dataset with anomalous data
if [ ! -e ./output/cad_run${run}_sol${sol}.mtz ]; then cad HKLIN1 $model HKLIN2 $data HKLOUT ./output/cad_run$
{run}_sol${sol}.mtz <<EOF  >./output/cad_run${run}_sol${sol}.log; fi

LABIN FILE 1 E1 = PHIC E2 = FOM
LABOUT FILE 1 E1 = PHIC E2 = FOM
CTYPEIN FILE 1 E1 = P E2 = W
LABIN FILE 2 E1 = DANO E2 = SIGDANO
LABOUT FILE 2 E1 = DANO E2 = SIGDANO
CTYPEIN FILE 2 E1 = D E2 = Q
END
EOF

#fft to get anomalous difference peaks

############
## cutoff1
if [ ! -e ./output/fft_run${run}_sol${sol}_cutoff${cutoff1}.log ]; then fft HKLIN ./output/cad_run${run}_sol$
{sol}.mtz MAPOUT ./output/fft_run${run}_sol${sol}_cutoff${cutoff1}.map <<EOF >./output/fft_run${run}_sol$
{sol}_cutoff${cutoff1}.log; fi
XYZLIM ASU
SCALE F1 1.0
#SCALE F2 1.0
RESOLUTION 100 $cutoff1
LABIN DANO=DANO SIG1 = SIGDANO PHI=PHIC W=FOM
END
EOF

#move map to model
if [ ! -e ./output/mapmask_run${run}_sol${sol}_cutoff${cutoff1}.log ]; then mapmask MAPIN ./output/fft_run$
{run}_sol${sol}_cutoff${cutoff1}.map MAPOUT ./output/mapmask_run${run}_sol${sol}_cutoff${cutoff1}.map XYZIN
./output/${pdb}.pdb <<EOF >./output/mapmask_run${run}_sol${sol}_cutoff${cutoff1}.log; fi
BORDER 20
END
```

```
EOF

#peakmax
if [ ! -e ./output/peakmax_run${run}_sol${sol}_cutoff${cutoff1}.log ]; then peakmax MAPIN ./output/mapmask_run$
{run}_sol${sol}_cutoff${cutoff1}.map XYZOUT ./output/peakmax_run${run}_sol${sol}_cutoff${cutoff1}.pdb XYZFRC
./output/peakmax_run${run}_sol${sol}_cutoff${cutoff1}.ha <<EOF >./output/peakmax_run${run}_sol${sol}_cutoff$
{cutoff1}.log; fi
THRESHOLD RMS 1.0
NUMPEAKS 50
OUTPUT BROOKHAVEN FRAC
RESIDUE WAT
ATNAME OW
CHAIN X
END
EOF

# grep for first peak
peak=""
peak=`grep "ATOM1 " ./output/peakmax_run${run}_sol${sol}_cutoff${cutoff1}.ha | awk '{print $6}'`

if [ "$peak" = "" ]; then
  peak="n/a"
fi

#print result
printf "%3s-%.2d %5s %6s %11s\n" $run ${sol} $peak $fom $cutoff1 >>tmp3${run}.txt

#############
## cutoff2
if [ ! -e ./output/fft_run${run}_sol${sol}_cutoff${cutoff2}.log ]; then fft HKLIN ./output/cad_run${run}_sol$
{sol}.mtz MAPOUT ./output/fft_run${run}_sol${sol}_cutoff${cutoff2}.map <<EOF >./output/fft_run${run}_sol$
{sol}_cutoff${cutoff2}.log; fi
XYZLIM ASU
SCALE F1 1.0
#SCALE F2 1.0
RESOLUTION 100 $cutoff2
LABIN DANO=DANO SIG1 = SIGDANO PHI=PHIC W=FOM
END
EOF

#move map to model
if [ ! -e ./output/mapmask_run${run}_sol${sol}_cutoff${cutoff2}.log ]; then mapmask MAPIN ./output/fft_run$
{run}_sol${sol}_cutoff${cutoff2}.map MAPOUT ./output/mapmask_run${run}_sol${sol}_cutoff${cutoff2}.map XYZIN
./output/${pdb}.pdb <<EOF >./output/mapmask_run${run}_sol${sol}_cutoff${cutoff2}.log; fi
BORDER 20
END
EOF

#peakmax
if [ ! -e ./output/peakmax_run${run}_sol${sol}_cutoff${cutoff2}.log ]; then peakmax MAPIN ./output/mapmask_run$
{run}_sol${sol}_cutoff${cutoff2}.map XYZOUT ./output/peakmax_run${run}_sol${sol}_cutoff${cutoff2}.pdb XYZFRC
./output/peakmax_run${run}_sol${sol}_cutoff${cutoff2}.ha <<EOF >./output/peakmax_run${run}_sol${sol}_cutoff$
{cutoff2}.log; fi
THRESHOLD RMS 1.0
NUMPEAKS 50
OUTPUT BROOKHAVEN FRAC
RESIDUE WAT
ATNAME OW
CHAIN X
END
EOF

# grep for first peak
peak=""
peak=`grep "ATOM1 " ./output/peakmax_run${run}_sol${sol}_cutoff${cutoff2}.ha | awk '{print $6}'`

if [ "$peak" = "" ]; then
  peak="n/a"
fi

#print result
printf "%3s-%.2d %5s %6s %11s\n" $run ${sol} $peak $fom $cutoff2 >>tmp3${run}.txt

#############
## cutoff3
if [ ! -e ./output/fft_run${run}_sol${sol}_cutoff${cutoff3}.log ]; then fft HKLIN ./output/cad_run${run}_sol$
{sol}.mtz MAPOUT ./output/fft_run${run}_sol${sol}_cutoff${cutoff3}.map <<EOF >./output/fft_run${run}_sol$
{sol}_cutoff${cutoff3}.log; fi
XYZLIM ASU
SCALE F1 1.0
#SCALE F2 1.0
RESOLUTION 100 $cutoff3
LABIN DANO=DANO SIG1 = SIGDANO PHI=PHIC W=FOM
END
EOF

#move map to model
if [ ! -e ./output/mapmask_run${run}_sol${sol}_cutoff${cutoff3}.log ]; then mapmask MAPIN ./output/fft_run$
```

```
{run}_sol${sol}_cutoff${cutoff3}.map MAPOUT ./output/mapmask_run${run}_sol${sol}_cutoff${cutoff3}.map XYZIN
./output/${pdb}.pdb <<EOF >./output/mapmask_run${run}_sol${sol}_cutoff${cutoff3}.log; fi
BORDER 20
END
EOF

#peakmax
if [ ! -e ./output/peakmax_run${run}_sol${sol}_cutoff${cutoff3}.log ]; then peakmax MAPIN ./output/mapmask_run$
{run}_sol${sol}_cutoff${cutoff3}.map XYZOUT ./output/peakmax_run${run}_sol${sol}_cutoff${cutoff3}.pdb XYZFRC
./output/peakmax_run${run}_sol${sol}_cutoff${cutoff3}.ha <<EOF >./output/peakmax_run${run}_sol${sol}_cutoff$
{cutoff3}.log; fi
THRESHOLD RMS 1.0
NUMPEAKS 50
OUTPUT BROOKHAVEN FRAC
RESIDUE WAT
ATNAME OW
CHAIN X
END
EOF

# grep for first peak
peak=""
peak=`grep "ATOM1 " ./output/peakmax_run${run}_sol${sol}_cutoff${cutoff3}.ha | awk '{print $6}'`

if [ "$peak" = "" ]; then
  peak="n/a"
fi

#print result
printf "%3s-%.2d %5s %6s %11s\n" $run ${sol} $peak $fom $cutoff3 >>tmp3${run}.txt

# sort the 3 cutoff lines and only use the best one.
sort -k2 -r tmp3${run}.txt >>result_sorted1_run${run}.txt
head -n 1 result_sorted1_run${run}.txt >> tmp${run}.txt
rm tmp3${run}.txt result_sorted1_run${run}.txt

done

# cleanup
rm output/*_run${run}_*

# process final result
sort -k2 -r tmp${run}.txt >>tmp2_run${run}.txt
echo "   run  peak    FOM  fft-cutoff" >result_run${run}.txt
cat tmp2_run${run}.txt >>result_run${run}.txt
rm tmp${run}.txt tmp2_run${run}.txt
```
